# The Influence of Light Exposure in Ambiance during Pregnancy in Maternal and Fetal Outcomes: An Experimental Study

**DOI:** 10.1055/s-0038-1675610

**Published:** 2018-11-14

**Authors:** Vitor Coca Sarri, Beatriz Maria Ferrari, Larissa Fernandes Magalhães, Paula Almeida Rodrigues, Almir Coelho Rezende, Marisa Afonso Andrade Brunherotti

**Affiliations:** 1 Department of Medicine, Universidade de Franca, Franca, SP, Brazil; 2 Department of Veterinary Medicine, Universidade de Franca, Franca, SP, Brazil; 3 Department of Physical Therapy, Universidade de Franca, Franca, SP, Brazil; 4 Department of Health Promotion and Medicine, Universidade de Franca, Franca, SP, Brazil

**Keywords:** circadian rhythm, pregnancy, embryonic and fetal development, light, ritmo circadiano, gravidez, desenvolvimento embrionário e fetal, luz

## Abstract

**Objective** The aim of this study is to evaluate whether exposure to different environmental lighting conditions affects the reproductive parameters of pregnant mice and the development of their offspring.

**Methods** Fifteen pregnant albino mice were divided into three groups: light/dark, light, and dark. The animals were euthanized on day 18 of pregnancy following the Brazilian Good Practice Guide for Euthanasia of Animals. Maternal and fetal specimens were measured and collected for histological evaluation. Analysis of variance (ANOVA) test was used for comparison of the groups considering *p* ≤ 0.05 to be statistically significant.

**Results** There was no significant difference in the maternal variables between the three groups. Regarding fetal variables, significant differences were observed in the anthropometric measures between the groups exposed to different environmental lighting conditions, with the highest mean values in the light group. The histological evaluation showed the same structural pattern of the placenta in all groups, which was within the normal range. However, evaluation of the uterus revealed a discrete to moderate number of endometrial glands in the light/dark and light groups, which were poorly developed in most animals. In the fetuses, pulmonary analysis revealed morphological features consistent with the transition from the canalicular to the saccular phase in all groups.

**Conclusion** Exposure to different environmental lighting conditions had no influence on the reproductive parameters of female mice, while the offspring of mothers exposed to light for 24 hours exhibited better morphometric features.

## Abstract (pt)

**Resumo:**

**Objetivo** Avaliar se a exposição a diferentes luminosidades no ambiente afeta parâmetros reprodutivos de camundongos fêmeas prenhas e o desenvolvimento de sua prole.

**Métodos** Foram selecionados para o estudo 15 camundongos fêmeas albinas prenhas. Os camundongos foram separados em grupos: luz/escuro, luz e escuro. As fêmeas foram eutanasiadas no 18° dia de gestação, seguindo as recomendações do Guia Brasileiro de Boas Práticas para a Eutanásia de animais. Tanto peças maternas como fetais foram mensuradas e coletadas para avaliação histológica. Foi utilizado o teste de Análise de variantes (Anova) para comparação dos grupos, considerando estatisticamente significativo o valor de *p* ≤ 0,05.

**Resultados** Ao comparar as variáveis maternas entre os três grupos, não foi encontrada diferença estatística significativa. Em relação às variáveis fetais, houve diferenças estatísticas entre as medidas de antropometria dos grupos submetidos a diferentes luminosidades do ambiente, com melhores valores médios no grupo luz. Histologicamente, a avaliação placentária evidenciou em todos os grupos o mesmo padrão estrutural, com todos dentro da normalidade. No entanto, a avaliação de úteros, tanto do grupo luz/escuro quanto do grupo luz, mostrou quantidade discreta a moderada de glândulas endometriais, com pouco desenvolvimento na maioria dos animais. Nos fetos, análise pulmonar evidenciou características morfológicas compatíveis com a transição da fase canalicular para sacular em todos os grupos.

**Conclusão** As exposições a diferentes luminosidades no ambiente não influenciaram nos parâmetros reprodutivos das fêmeas, entretanto, a ninhada das mães que receberam luz em todo período apresentou melhores medidas morfométricas.

## Introduction

Light in the environment interferes with the biological function of different systems, and circadian rhythm activities are related to light variation. The periods of sleep and wakefulness are directly associated with the circadian rhythm, and the restriction of nocturnal sleep during pregnancy can affect hypothalamic hormones, plasma cortisol, and body weight.^1^ ^2^

The sleep-wake cycle may be altered by working shift, and some health issues, such as reproductive success, mating and pregnancy problems are related to working at night or shiftwork. Alterations in the biological rhythm caused by shiftwork are intimately linked to changes in the female hormonal cycle, and consequently in reproductive function.^2^ ^3^ Melatonin, an indolamine produced by the pineal gland, plays a key role in the regulation of the circadian rhythm. This hormone is secreted during the night and its function in mammals is to mediate signals of darkness.^4^ Environmental light is the most important factor for the regulation of melatonin synthesis, responsible for circadian rhythm and its secretion. Exposure to light at night acutely inhibits the synthesis of melatonin; however, darkness does not stimulate its production.^5^ It should be noted that the presence of light, even of low intensity (50–300 lux) as found in residences, can inhibit the production of melatonin in humans.

Variations in serum melatonin levels are closely related to ovulation disorders and the function of melatonin in the female ovarian cycle is associated with steroidogenesis.^6^ Melatonin resulting from the production related to the circadian rhythm is transferred from the mother to the fetus through the placenta or maternal milk.^7^ Thus, exposure to light and the consequent deregulation of the maternal circadian rhythm can possibly cause repercussions for the fetus.

The question proposed using an experimental animal model is whether different times of exposure to artificial light environmental during pregnancy causes changes in morphological and histological parameters of mother and fetus. There are no evidences yet in literature to demonstrate the effect comparing luminosity differences in the ambiance throughout the gestational period. Therefore, the objective of the present study is to evaluate whether different environmental lighting conditions affect the reproductive parameters of pregnant females and the development of their offspring.

## Methods

This study was conducted at the Animal House of the Universidade de Franca within the Maternal-Infant project of the Laboratory of Health Promotion Strategies. The study was approved by the Ethics Committee on Animal Use of the Universidade de Franca (Protocol number 015/15). Fifteen female albino Swiss Webster mice (*Mus musculus*) obtained from the Animal House of the Universidade de São Paulo (USP, in the Portuguese acronym) in Ribeirão Preto were selected for this study. They were 90 days of age and weighed ∼ 40 g. The animals were kept under the following conditions: constant air renewal, ambient temperature of 22 ± 2°C, and humidity of 50%. Water and ration were available *ad libitum*. The females selected for this study were mated and divided into three groups: 1) light group, consisting of five pregnant mice kept in the presence of light for 24 hours; 2) dark group, consisting of five pregnant mice kept in the dark for 24 hours; 3) light/dark group, consisting of five pregnant mice kept under a 12/12-hours light/dark cycle, with lights on from 6 _AM_ to 6 _PM_.

The animals were mated at a proportion of one male per female, always in the morning (7 AM). Mating was confirmed by the inspection of the vaginal plug, always 2 hours after the female-male exposure, and according to the presence of seminal fluid in the vagina, the test was considered positive and considered day zero of pregnancy. The mice selected for the study were nulliparous.

The animals were kept in plastic cages (2 animals/cage) for a period of 18 days. Animals from the light/dark group (12 hours light and 12 hours dark) were maintained on a normal day/night cycle. The light group was exposed to constant cold light in the room for 24 hours. Animals of the dark group were kept in a completely dark room for 24 hours. For this purpose, the windows were covered with a double layer of brown paper, as were the shelves containing the cages. A FoxLux Timer (FoxLux Ltda., Pinhais, PR, Brazil) was used for light control in a room with light-beige colored walls measuring ∼ 11 m^2^, with a rail containing two fluorescent lamps and slate floor. The light intensity on a scale of 2,000 was: center of the room (2,000:180 lux), back (2,000:56 lux), and front (2,000:65 lux). The experiments were conducted in the center of the room.

The animals were killed following the Brazilian Good Practice Guide for Euthanasia of Animals.^8^ Female mice were killed by intraperitoneal injection of thiopental (150 mg/kg). Their offspring were anesthetized by hypothermia (immersion in ice for 20 minute), followed by decapitation with a sharp blade. Females were euthanized on day 18 of pregnancy. The fetus and placenta were immersed in saline 0.9% and transferred to absorbent paper towels for the complete removal of fluid or any type of residue before measurements to avoid false results. The fetuses collected were weighed on a MARTE AL500 high-precision scale (Marte Científica, São Paulo, SP, Brazil), and their length was measured with a caliper (millimeter scale). The fetal skull, chest and lungs, and maternal uterus and placenta were fixed in 10% paraformaldehyde for 24 hours and transferred to alcohol 70% before embedding them in paraffin. Routine staining with hematoxylin and eosin (H&E) was used for histological analysis.

The results were compared between groups using the analysis of variance (ANOVA) test, followed by the Tukey test. A *p*-value ≤ 0.05 was considered statistically significant. The BioStart 5.0 (AnalystSoft Inc., Walnut, Canada) program was used for statistical analysis of the data.

## Results

The results have demonstrated that the difference of luminosity in the ambiance seems to have no influence in the female reproductive parameters, however, they suggest that it has influence on the fetus morphometric parameters. Table 1 shows the maternal variables. No significant differences were observed between groups. Analysis of the weight evolution of females during pregnancy showed a similar average weight gain and final weight in the three groups, with no statistically significant difference. However, weight gain was lower in females exposed to light for 24 hours compared with the other two groups, but no statistic difference was observed in weight gain. The same was observed for litter size, with no significant difference between the three groups. However, a smaller litter was found in the group submitted to light deprivation during the experiment (average of 9.2 ± 4.2 offspring per litter). The number of resorptions did not differ significantly between groups. However, no resorption was observed in the group exposed to light for 24 hours (Table 1). Placental and uterine weights were also similar in the groups. The same trend was observed for uterine weight (Table 1).

**Table 1:** Characteristics of pregnant mice sample

	Light (*n* = 5)	Dark (*n* = 5)	Light/dark (*n* = 5)	*p*-value
**Final weight (g)**				
Average	63.0	65.7	67.1	0.71
SD	6.6	10.4	5.0	
CV (%)	10.4	15.9	7.4	
**Litter size (n)**				
Average	12.0	9.2	13.6	0.15
SD	2.7	4.2	2.9	
CV (%)	22.8	46.3	21.8	
**Uterine weight (g)**				
Average	1.7	1.5	1.7	0.57
SD	0.08	0.34	0.24	
CV (%)	4.8	22.8	14.4	
**Resorption (n)**				
Mean	0	2.4	0.6	0.26
SD	0	3.9	0.5	
CV (%)		98.0	91.2	
**Placental weight (g)**				
Average	1.6	1.2	1.6	0.35
SD	0.4	0.6	0.3	
CV (%)	29.2	48.5	24.0	
**Estimated litter (n)**				
Average	12.0	11.6	14.2	0.20
SD	2.7	0.8	2.7	
CV (%)	22.8	7.7	19.5	

Abbreviations: CV, coefficient of variation; SD, standard deviation.

Analysis of variance followed by Tukey test, with *p* < 0.05 indicating statistical significance.

**Fig. 1:**
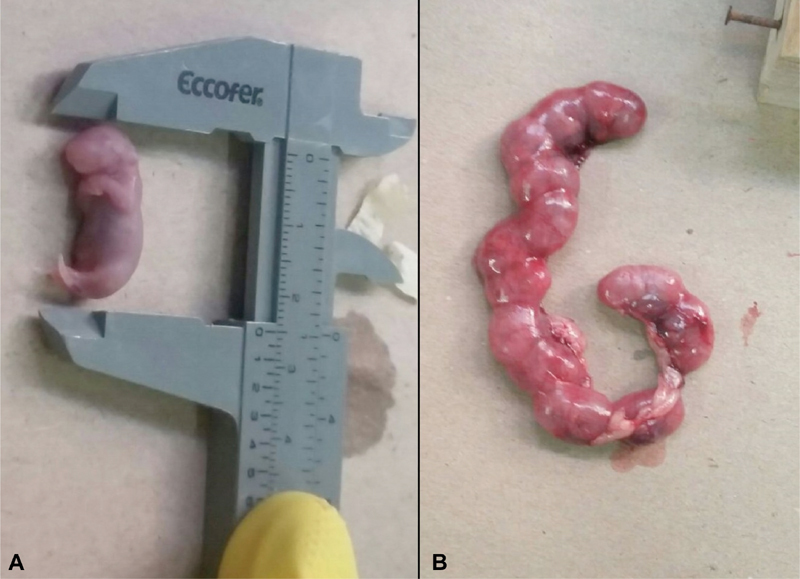
(**A**) Photograph of the morphometric evaluation of a mouse fetus of the control group. (**B**) Image of the uterus of a control mouse.

Table 2 shows significant differences in the fetal variables between the groups exposed to different environmental lighting conditions. Average fetal length (Fig. 1) was significantly higher in the light group compared with the dark group (*p* < 0.05) and light/dark group (*p* < 0.01). Fetal weight was also higher in the light group compared with the other groups (*p* < 0.01). On the other hand, no significant difference in weight was observed between the dark and light/dark groups. Analysis of fetal cranial measures showed better average growth values in the light group. The anteroposterior and laterolateral lengths of the skull differed significantly between light and light/dark groups (*p* < 0.01) and between dark and light/dark groups (*p* < 0.01), while no difference was found between light and dark groups. Average skull weight was significantly higher in the light group compared with the dark group (*p* < 0.05) and the light/dark group (*p* < 0.01), also, there was a difference between the dark and light/dark groups (*p* < 0.05; Table 2). Similarly, the average chest variables tended to be higher in the light group (Table 2). The mean superoinferior diameter of the chest was significantly greater in comparison to the light and light/dark groups (*p* < 0.01) and to the dark and light/dark groups (*p* < 0.05). The average laterolateral diameter was similar in the three groups.

**Table 2:** Morphometric variables of the 134 fetuses of 15 albino Swiss Webster mice (*Mus musculu* *s*)

Variable	Light (*n* = 60)	Dark (*n* = 46)	Light/dark (*n* = 68)
**Length (mm)**			
Average	24.5*	22.8	22.2
SD	1.1	1.9	4.4
CV (%)	4.6	8.4	20.1
**Weight (g)**			
Average	1.4*	1.2	1.1
SD	0.08	0.14	0.33
CV (%)	5.6	11.9	28.3
**Anteroposterior skull (mm)**			
Average	11.2	11.1	10.3**
SD	0.5	0.5	1.2
CV (%)	5.1	5.2	11.6
**Laterolateral skull (mm)**			
Average	7.4	7.1	6.7**
SD	0.5	0.4	1.0
CV (%)	7.3	6.0	16.0
**Skull weight (g)**			
Average	0.33¥	0.30ϕ	0.28
SD	0.06	0.3	0.04
CV (%)	18.3	9.8	16.8
**Superoinferior thoracic diameter (mm)**			
Average	10.1	10.0	9.5**
SD	0.8	0.7	1.3
CV (%)	7.9	7.7	14.3
**Laterolateral thoracic diameter (mm)**			
Average	8.1β	7.7α	8.5
SD	0.5	0.6	0.9
CV (%)	6.2	7.8	11.0
**Thorax weight (g)**			
Average	0.47*	0.40	0.39
SD	0.05	0.04	0.05
CV (%)	11.9	11.9	14.7

Abbreviations: CV, coefficient of variation; mm, millimeters; SD, standard deviation.

Analysis of variance followed by Tukey test, with *p* < 0.05 indicating statistical significance.

*: *p* < 0.01 light group vs dark and light/dark groups.

**: *p* < 0.01 light/dark group vs light and dark groups.

¥: *p* < 0.05 light group vs dark group, and *p* < 0.01 light group vs light/dark group.

ϕ: *p* < 0.05 dark group vs light/dark group.

β: *p* < 0.01 light group vs dark group, and *p* < 0.05 light group vs light/dark group.

α: *p* < 0.01 dark group vs light/dark group.

**Fig. 2:**
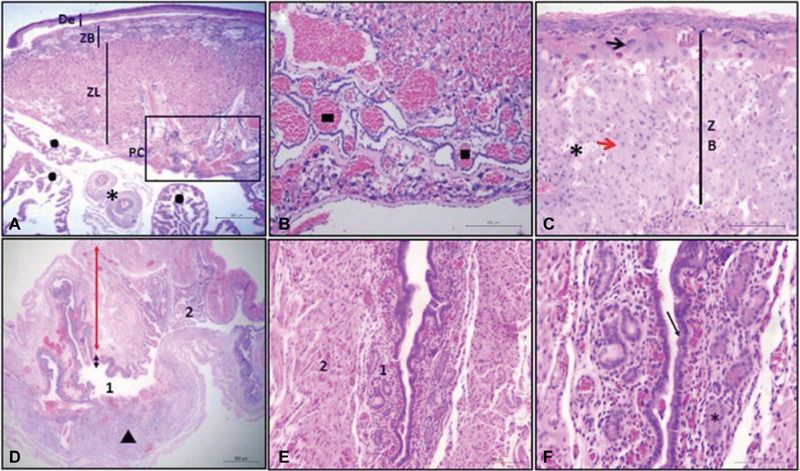
(**A**) Photomicrograph of the hemotrichorial placenta in a mouse of the light/dark group. De: decidua; ZB: basal zone; ZL: labyrinth zone; PC: chorionic placenta; •: yolk sac; * umbilical cord vessels. H&E, 2.5x. (**B**) Photomicrograph of the hemotrichorial placenta in a mouse of the light/dark group. ▪: Chorionic vessels in the chorionic plate. H&E,10x. (**C**) Photomicrograph of the hemotrichorial placenta in a mouse of the light/dark group. Note the three types of cells in the basal layer: trophoblast giant cells (black arrow) separating the basal zone (ZB) and decidua (De); glycogen cells (asterisk), and spongiotrophoblast cells (red arrow). H&E., 10x. (**D**) Photomicrograph of the uterus in a mouse of the light/dark group. 1: Uterine lumen where the dark line indicates the endometrium and the red line the myometrium; ▴: placental tissue; 2: uterine tube. H&E., 2.5x. (**E**) Photomicrograph of the uterus in a mouse of the light/dark group. 1: endometrium with a moderate number of glands. Note the eosinophilic content in the lumen of some glands. 2: Myometrium. H&E., 2.5x. (**F**) Photomicrograph of the uterus in a mouse of the light/dark group. Arrow: simple cylindrical epithelium; *: eosinophilic content in some glands. H&E, 20x.

All placentas and uteruses were submitted to histological analysis. Placental evaluation revealed the same normal structural pattern in all groups, showing cellular features typical of the species (Fig. 2). Evaluation of the uteruses showed a discrete to moderate number of endometrial glands in the light/dark and light groups, which were poorly developed in most animals, except for one animal in the light group that presented well-developed endometrial glands. Neovascularization in the lamina propria was observed in all fragments (Fig. 2).

**Fig. 3:**
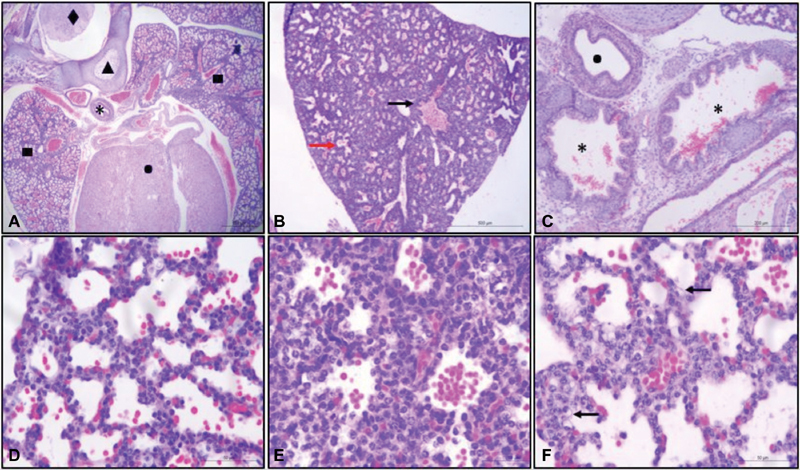
(**A**) Photomicrograph of a cross-section of the fetal chest in the light/dark group. ▪: Lung tissue; •: heart; *: esophagus; ▴: vertebral body; ♢: spine. H&E, 2.5x. (**B**) Photomicrograph of the fetal lung in the light/dark group. Black arrow: bronchioles; red arrow: expansion of the alveolar sacs containing red blood cells and a moderate amount of mesenchyme. H&E, 5x. (**C**) Photomicrograph of the fetal lung in the light/dark group. *: Bronchioles; •: esophagus. (**D**) Photomicrograph of the fetal lung in the light/dark group. H&E, 20x. (**E**) Photomicrograph of the fetal lung in the light/dark group. Note the difference in the amount of mesenchyme. H&E, 40x. (**F**) Photomicrograph of the fetal lung in the light/dark group. Arrows: type II pneumocytes.

Histological parameters of the chest and lungs of the fetuses were evaluated. Thorax assessment revealed the presence of skin, muscle, cartilage, vertebral bodies, spine, esophagus, trachea, thymus, heart, and lung in all groups. Pulmonary analysis showed morphological features consistent with the transition from the canalicular to the saccular phase in all animals. Only one animal from the light group exhibited tubuloacinar structures in the absence of alveolar expansion and undifferentiated septal cells, findings suggestive of the pseudoglandular phase (Fig. 3).

## Discussion

Considering the influence of environmental light, that is, light/dark cycle, on the biological system, the findings for animals submitted to a light/dark period are in concordance with the literature regarding litter size and average final female weight. The litter of this study was composed by 113 pups, with an average of 13.6 pups per female. An average of 8 to 10 pups per litter was reported in a study investigating the control of reproduction in animal houses conducted in 2002.^8^

In the present study, the best average maternal variables were found for the light/dark group. In this group, daytime and night-time periods were simulated, which influences in an expected way the normal circadian rhythm of an individual who performs his/her activities during the day and rests at night. The secretion of hormones and melatonin follows the biological rhythm and does not affect the biological activities of the organism.

The weight gain of females was higher in the light/dark group compared with the two other groups, since the animals' normal routine was maintained in this group, with melatonin secretion following the normal rhythm of the organism. The biological rhythm of the animals was maintained close to normal. On the other hand, and in contrast to the literature, a comparison of weight gain between the light and dark groups showed a higher weight gain in animals deprived of light for 24 hours. Melatonin deprivation or a reduction in its production has been shown to induce higher weight gain and can possibly cause obesity.^9^

No histological placental alterations were observed in any of the groups, suggesting that the exchange of nutrients after the placenta formation in pregnant mice was not affected. On the other hand, histological analysis of the uteruses showed a reduction in the number of endometrial glands in the light/dark and light groups. These glands are necessary to provide adequate nutrition to the embryo, especially early on the pregnancy, when the placental circulation is not fully established. Despite the alterations in endometrial glands, the light/dark group gave birth to the largest litter of the experiment.

The largest number of resorptions was observed in the dark group. Resorptions are defined as the cessation of embryo development and are found after removal of the uterus. They resemble the placenta but are smaller. Resorption can occur if the female is exposed to a male pheromone that differs from the mating pheromone within 24 hours after copulation.^8^ The environment of our study was controlled to avoid such exposure. Thus, the resorptions found were due to nutritional or structural deficits caused by the deregulation of the circadian rhythm of the animals.

Melatonin receptors are found in the pineal gland, and also in other organs such as the reproductive organs of humans and animals. Melatonin exerts action on the ovaries and uterus and is also involved in placental implantation after mating.^10^ ^11^ Light exposure for short periods is unable to cause changes in maternal development. Alterations have been reported when female mice are exposed to light for long periods before mating.^11^ In our study, females were exposed after mating, a fact that may explain the normal development of pregnancy.

Alterations resulting from light exposure before mating, particularly morphological changes, are caused by melatonin. These alterations mainly occur in the ovarian tissue, leading to the development of polycystic ovaries in some females. In the uterus, the changes are related to hypertrophy of the endometrial epithelium.^11^

Statistical analysis of fetal parameters showed a better development of almost all parameters in the group exposed to light for 24 hours. This might be explained by the longer period of maternal cortisol secretion. Since cortisol is regulated by the circadian rhythm,^12^ the peak production of this hormone depends on the presence of light, and lower concentrations are thus observed when the animal is deprived of light. The passage of maternal cortisol to the fetus throughout pregnancy is well-established.^13^ Considering this maternal-fetal exchange, this pro-catabolic hormone can be related to greater structural development of the offspring, since the fetuses would be more exposed to its effects due to higher maternal secretion.

Microscopic analysis of the fetal specimens in all groups did not reveal major structural alterations. Except for one animal of the light group that exhibited a delayed pulmonary development, the parameters were similar in the remaining 133 fetuses. This finding suggests that the exposure to different lighting conditions and the significant morphometric alterations were not sufficient to cause changes at the cellular level in the animals studied.

With these results, we can consider that shift factors must have attention to the pregnant women health as an employment risk factor. Thus, expanding to bigger interests in investigation in ambiance and health investigation, providing action plans for prevention and health promotion.

## Conclusion

The present results show that exposure to different lighting conditions during pregnancy did not influence female reproductive parameters, while pups exposed to light throughout pregnancy exhibited better morphometric measures. However, variations in luminosity had no negative influence on the pregnancy of mice.
